# Integrated network toxicology, bioinformatics, and molecular docking reveal the potential molecular mechanisms linking bisphenol A to glioma progression

**DOI:** 10.3389/fbinf.2026.1857455

**Published:** 2026-07-10

**Authors:** Lei Miao, Fengwei Hou

**Affiliations:** Heze Medical College, Heze, Shandong, China

**Keywords:** bisphenol A, glioma, molecular docking, network toxicology, STAT3/AKT1 signaling

## Abstract

Glioma is a highly aggressive central nervous system malignancy with poor clinical outcomes, and increasing attention has focused on whether environmental endocrine-disrupting chemicals contribute to its progression. This study aimed to systematically investigate the molecular mechanisms linking bisphenol A (BPA) to glioma using an integrated network toxicology and bioinformatics strategy. BPA-related targets were collected from public databases and intersected with glioma-associated genes to identify shared targets. A protein–protein interaction network was then constructed to screen hub genes, followed by transcriptomic validation using the GSE41031 dataset. Functional enrichment analyses were performed to characterize the biological processes and signaling pathways involved, and molecular docking was used to assess the binding potential of BPA with representative core targets. A total of 696 common targets were identified between BPA and glioma. Network analysis highlighted 20 hub genes, among which STAT3, AKT1, TNF, IL6, and TP53 showed the highest topological importance. Most hub genes were significantly dysregulated in glioma stem cells relative to normal neural stem cells. Enrichment analyses indicated that the shared targets were mainly associated with oxidative stress, hypoxia, xenobiotic response, steroid hormone signaling, apoptosis, focal adhesion, and the PI3K-Akt pathway. Molecular docking suggested moderate predicted binding compatibility between BPA and the five selected hub proteins. These *in silico* findings suggest that BPA-related targets are potentially associated with glioma-relevant inflammatory, stress-response, and survival-related signaling networks. This study provides a systems-level framework for understanding the potential contribution of BPA to glioma biology and identifies candidate molecular targets for future mechanistic and translational investigations.

## Graphical Abstract

**Figure:**
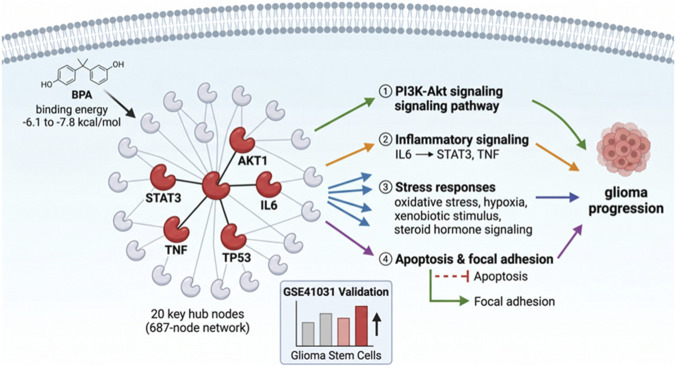

## 1 Introduction

Glioma remains one of the most devastating malignancies of the central nervous system because of its diffuse infiltration, marked molecular heterogeneity, therapeutic resistance, and persistently poor survival, especially in high-grade disease (Zhang R. et al., 2026; Ormond et al., 2026). Even as molecular classification and precision oncology continue to refine diagnosis and treatment, the prognosis of aggressive glioma is still unsatisfactory, highlighting the need to identify previously underappreciated determinants of tumor initiation and progression. At the same time, growing concern has focused on the health consequences of long-term exposure to endocrine-disrupting chemicals in modern environments (Pinson et al., 2025; Singh, 2024; Zeng et al., 2023). Among them, bisphenol A (BPA), a ubiquitous industrial compound used in polycarbonate plastics and epoxy resins, has attracted sustained attention because human exposure is widespread and BPA can interfere with hormone signaling, redox balance, inflammation, and cell survival pathways that are also central to cancer biology (Li et al., 2016; Brandolino et al., 2025; Cp et al., 2025).

Recent studies suggest that the biological effects of BPA extend far beyond classical endocrine tissues (Wolstenholme et al., 2011; Jirtle, 2025). Increasing evidence indicates that BPA can affect the nervous system, alter oxidative stress responses, modulate inflammatory signaling, and disrupt transcriptional programs relevant to brain homeostasis and disease (Li et al., 2023). These observations are particularly noteworthy in glioma, a tumor type whose progression is tightly linked to dysregulated proliferation, apoptosis resistance, hypoxia adaptation, immune signaling, and PI3K/AKT-associated oncogenic networks (Zhang W. et al., 2025). Despite increasing attention to endocrine disruptors and brain health, three major gaps remain. First, the target landscape through which BPA may influence glioma has not been systematically mapped at the whole-network level. Second, most existing studies have emphasized isolated cellular observations, whereas the relationships among BPA-responsive targets, glioma-associated genes, hub regulators, and enriched signaling pathways remain poorly integrated. Third, there is still limited effort to combine target prediction, interaction network analysis, transcriptomic validation, and structural binding assessment within a unified framework for BPA-induced glioma.

The study design integrates BPA-related targets and glioma-associated genes, identifies their intersection, prioritizes hub genes through protein–protein interaction topology, verifies the expression relevance of candidate core genes using an external GEO dataset, and further interrogates the biological functions and putative ligand–target interactions of representative hubs. This progression directly addresses the need for a mechanism-oriented, multi-step analytical framework for understanding how BPA may participate in glioma biology. In this study, we aimed to systematically elucidate the potential molecular basis by which BPA may contribute to glioma progression using an integrated network toxicology strategy combined with bioinformatics validation and molecular docking. We hypothesized that BPA is associated with a defined subset of glioma-relevant targets and that these targets converge on key oncogenic and inflammatory signaling modules involved in cellular stress responses, apoptosis regulation, and tumor-related pathway remodeling. To test this hypothesis, we constructed a shared target landscape for BPA and glioma, identified central hub genes, examined their transcriptional relevance in an independent dataset, characterized enriched Gene Ontology and Kyoto Encyclopedia of Genes and Genomes pathways, and evaluated the binding potential of BPA toward representative core proteins.

By clarifying these molecular connections, this work seeks to provide a more coherent mechanistic basis for understanding the possible contribution of environmental BPA exposure to glioma-related pathobiology, while also offering candidate core targets and intervention-relevant signaling axes for future experimental and translational studies.

## 2 Methods

### 2.1 Collection of BPA-related targets

The chemical information of bisphenol A (BPA), including its two-dimensional structure, molecular formula, and canonical SMILES notation, was obtained from the PubChem database. To comprehensively identify potential BPA-associated targets, multiple public databases with complementary prediction strategies were queried, including PharmMapper, SwissTargetPrediction, and the Comparative Toxicogenomics Database (CTD). PharmMapper was used for reverse pharmacophore mapping-based target prediction, SwissTargetPrediction was used to infer candidate targets according to chemical similarity and known ligand–protein interactions, and CTD was used to retrieve curated chemical–gene associations supported by toxicological evidence.

All BPA-related targets collected from these databases were merged into a single dataset. Duplicate entries were removed after standardization, and target names were normalized using the UniProt database to ensure consistency in official gene symbols and species annotation. Only human-related targets were retained for downstream analyses. The final non-redundant target set was defined as the BPA-related target library and used for subsequent intersection analysis with glioma-associated genes. An inclusive target collection strategy was adopted to maximize coverage of potential BPA-responsive and glioma-associated genes at the exploratory stage. Therefore, the overlapping targets were regarded as a preliminary candidate pool rather than a definitive BPA–glioma-specific signature. Subsequent PPI analysis, hub-gene screening, transcriptomic validation, functional enrichment, and molecular docking were used to prioritize biologically relevant candidate targets from this broad overlap.

### 2.2 Collection of glioma-related targets

Glioma-related genes were collected from GeneCards and the Online Mendelian Inheritance in Man (OMIM) database by using the keyword “glioma.” GeneCards was used to retrieve genes associated with glioma based on integrated relevance scores derived from genomic, transcriptomic, functional, and literature evidence, whereas OMIM was used to identify genes with established disease relevance from curated genetic and clinical records.

To improve specificity while maintaining broad coverage, screening criteria were applied during data collection. In GeneCards, genes with a relevance score ≥1 were retained to enrich for targets with meaningful disease association. In OMIM, entries annotated as disease-related or pathogenic were included. The results from the two databases were integrated, and duplicate genes were removed to construct a unified glioma-related target dataset. Gene symbols were checked and standardized to official human gene names before subsequent analysis.

### 2.3 Visualization of “BPA-glioma” target interactions

The BPA targets and glioma-associated targets were mapped in a Venn diagram using the VennDiagram package in R.

### 2.4 Construction of the protein–protein interaction network and screening of hub genes

The intersecting gene set was imported into the STRING database to construct a protein–protein interaction (PPI) network. The organism was restricted to *Homo sapiens* to ensure biological relevance to human disease. A high-confidence interaction threshold was applied to reduce noise and improve network reliability. Both direct and indirect protein associations supported by experimental evidence, curated databases, co-expression, and computational prediction were considered within the STRING framework.

The resulting PPI network data were exported in TSV format and then imported into Cytoscape software for visualization and topological analysis. Within Cytoscape, the CytoHubba plugin was used to identify key hub genes that may play central regulatory roles in the common target network. The Matthews correlation coefficient (MCC) algorithm was selected as the ranking method because it is widely regarded as a robust approach for identifying essential nodes in complex biological networks. Genes were ranked according to their MCC scores, and the top 20 genes were defined as hub genes for subsequent validation and interpretation. The MCC algorithm was used to rank hub genes because it is widely applied in CytoHubba for identifying essential nodes in complex biological networks. The top 20 MCC-ranked genes were selected as hub genes to retain the most central and biologically interpretable targets while maintaining a manageable number of candidates for downstream validation and molecular docking. This cutoff was used as a prioritization strategy rather than an absolute biological threshold.

### 2.5 Validation of core gene expression using GEO datasets

To validate the reliability of the identified core genes, gene expression data were retrieved from the Gene Expression Omnibus (GEO) database. The GSE41031 dataset was selected for validation analysis. This dataset contained glioma stem cell samples and normal neural stem cell control samples. The expression levels of the core genes between the disease group and the control group were compared using Student’s t-test. Differences with *P* < 0.05 were considered statistically significant. The results were visualized as bar charts.

### 2.6 Gene Ontology (GO) and Kyoto Encyclopedia of Genes and Genomes (KEGG) enrichment analyses

To delineate the mechanism of BPA-induced glioma, intersected targets were converted to Entrez IDs *via* the org. Hs.eg.db R package. These curated identifiers underwent functional enrichment analysis implemented in clusterProfiler, with GO term annotation and KEGG pathway mapping performed concurrently (Wang et al., 2025). Significantly enriched terms (p-value and q-value <0.05) were filtered, yielding the top 10 GO processes and KEGG pathways ranked by ascending q-value (FDR-corrected significance). Result visualization integrated enrichplot and ggplot2 for multidimensional interpretation.

### 2.7 Molecular docking validation of BPA-hub target interactions

Molecular docking was employed to characterize binding mechanisms between Bisphenol A and glioma-associated hub targets, predicting binding modes and affinity energies through computational simulations. Protein structures (receptors) and the BPA ligand were prepared as follows: 1) BPA’s isomeric SMILES was curated from PubChem, 2) Hub target PDB files were acquired *via* UniProt IDs using PDB’s advanced search, and 3) The CB-Dock2 platform automated ligand/protein pre-processing including structural optimization (energy minimization, hydrogen addition) and solvent shell removal (explicit water elimination). Docking simulations identified optimal binding sites based on minimal Vina scores (≤-6.0 kcal/mol indicating strong binding). Docking validation was performed by re-docking the native ligands extracted from each PDB structure, achieving RMSD values <2.0 Å, confirming the reliability of the docking protocol. During docking, ligand flexibility was handled by allowing full torsional freedom for all rotatable bonds of BPA, whereas receptor side chains within 5 Å of the binding site were kept flexible using the CB-Dock2 default settings. Binding affinities were estimated with the Vina scoring function, expressed as binding free energy (ΔG, kcal/mol).

## 3 Results

### 3.1 Procurement of targets

**FIGURE 1:**
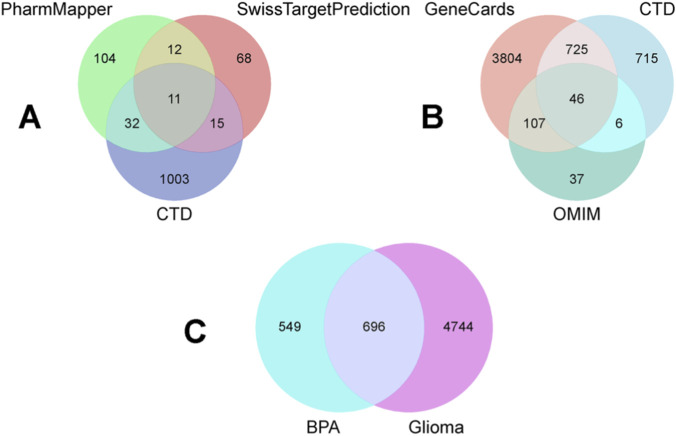
Target collection for BPA **(A)**. Target collection for Glioma **(B)**. Venn diagram showing intersecting targets between BPA and Glioma **(C)**.

To investigate the potential molecular relationship between bisphenol A (BPA) and glioma, BPA-associated targets and glioma-related genes were first collected from multiple public databases and then integrated after removal of duplicate entries. A total of 1,245 BPA-related targets were identified from PharmMapper, SwissTargetPrediction, and CTD (Figure 1A). For glioma, 4,682 targets were retrieved from GeneCards, 1,492 from CTD, and 196 from OMIM. After consolidation and deduplication, 5,440 unique glioma-related targets were obtained (Figure 1B).

The overlap analysis between the BPA target set and the glioma gene set identified 696 common targets (Figure 1C). These shared genes represent a preliminary candidate target pool through which BPA may be associated with glioma-related biological processes. The relatively large number of intersecting genes suggests that BPA may be associated with broad stress-response and cancer-related molecular networks; however, this overlap should be interpreted cautiously because it may include nonspecific or low-confidence targets and should not be regarded as evidence of a single isolated pathway.

### 3.2 PPI network construction and hub gene screening

To further clarify the biological relationships among the 696 intersecting targets, a protein–protein interaction (PPI) network was constructed using the STRING database. After excluding disconnected nodes, the resulting network consisted of 687 nodes and 18,935 edges, indicating dense interactions among the common targets. The average node degree was 28, suggesting that many of the intersecting genes were functionally interconnected and may cooperate in key pathogenic processes.

The PPI network was imported into Cytoscape for visualization and topological analysis. The CytoHubba plugin was then used to identify key regulatory genes within the network, and the Matthews correlation coefficient (MCC) algorithm was applied to rank nodes according to their centrality and potential biological importance. The top 20 hub genes were identified as STAT3, AKT1, TNF, IL6, TP53, JUN, NFKB1, BCL2, ALB, PPARG, MTOR, CASP3, CTNNB1, MMP9, BCL2L1, MYC, TGFB1, PTEN, ESR1, and CCND1.

**FIGURE 2:**
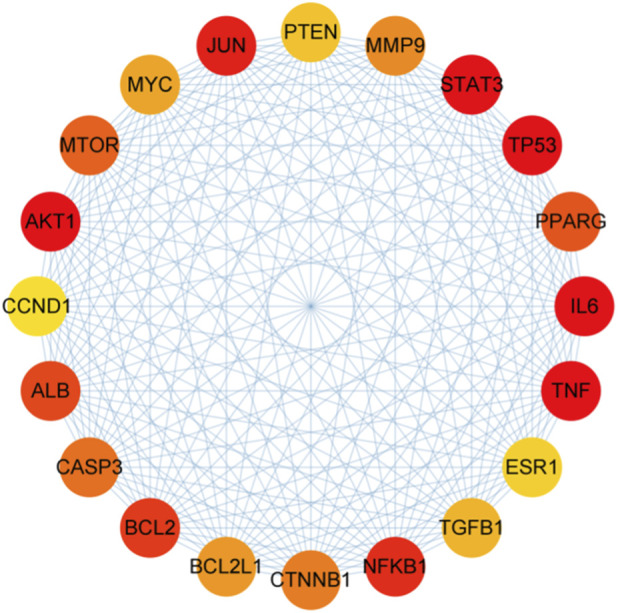
Core genes identified using the MCC algorithm. The network diagram represents complex interactions among 20 core genes, highlighting their functional connections. Among these STAT3, AKT1, TNF, IL6 and TP53 exhibit the highest MCC values, represented by redder node colors in the diagram.

Among these hub genes, STAT3, AKT1, TNF, IL6, and TP53 showed the highest MCC scores and occupied the most central positions in the network. These genes are known to participate in inflammation, survival signaling, cell cycle regulation, apoptosis, and tumor progression, suggesting that they may serve as major molecular nodes linking BPA exposure to glioma development. The network distribution and relative importance of these core targets are shown in Figure 2.

### 3.3 Validation of hub gene expression in the GSE41031 dataset

To assess whether the hub genes identified by network analysis were also transcriptionally relevant in a glioma-related biological context, the GSE41031 dataset from the GEO database was used for validation. This dataset included three glioma stem cell samples and three normal neural stem cell samples. The expression profiles of the 20 hub genes were compared between the two groups using Student’s t-test.

In the GSE41031 dataset, 15 of the 20 hub genes were significantly differentially expressed between glioma stem cells and normal neural stem cells. Most dysregulated genes showed higher expression in glioma stem cells than in normal neural stem cells, supporting the transcriptional relevance of the network-derived hub genes in a glioma stem-like cellular context (P < 0.05). In general, the majority of these genes showed higher expression levels in the glioma stem cell group than in the control group, indicating that the network-derived core targets are closely associated with the malignant phenotype. This finding supports the reliability of the PPI-based screening strategy and suggests that these hub genes are not merely computational artifacts, but are biologically relevant in glioma-associated cellular systems.

**FIGURE 3:**
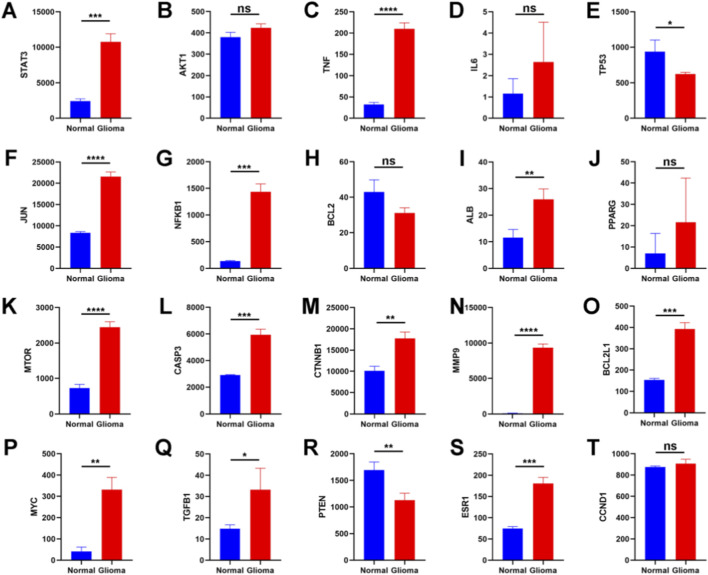
Differential expression profiles of 20 hub genes in the GSE41031 dataset. **(A)** STAT3; **(B)** AKT1; **(C)** TNF; **(D)** IL6; **(E)** TP53; **(F)** JUN; **(G)** NFKB1; **(H)** BCL2; **(I)** ALB; **(J)** PPARG; **(K)** MTOR; **(L)** CASP3; **(M)** CTNNB1; **(N)** MMP9; **(O)** BCL2L1; **(P)** MYC; **(Q)** TGFB1; **(R)** PTEN; **(S)** ESR1; **(T)** CCND1. *p < 0.05; **p < 0.01; ***p < 0.001; ****p < 0.0001; ns: not significant.

Notably, TP53 and PTEN showed lower expression in glioma stem cells than in normal neural stem cells, whereas BCL2 also showed a lower but non-significant trend. In addition, AKT1, IL6, PPARG, and CCND1 did not reach statistical significance. These results may reflect the complexity of hub-gene regulation in glioma and indicate that network centrality does not necessarily correspond to uniform transcriptional upregulation. Overall, the expression validation provided independent transcriptomic support for the involvement of the identified hub genes in glioma biology. The detailed differential expression profiles are shown in Figure 3.

### 3.4 GO functional enrichment analysis of intersecting targets

To explore the biological functions represented by the 696 common targets, Gene Ontology (GO) enrichment analysis was performed, including the biological process (BP), molecular function (MF), and cellular component (CC) categories. The results revealed that the intersecting genes were significantly enriched in multiple stress-response, inflammatory, and tumor-related functional modules.

**FIGURE 4:**
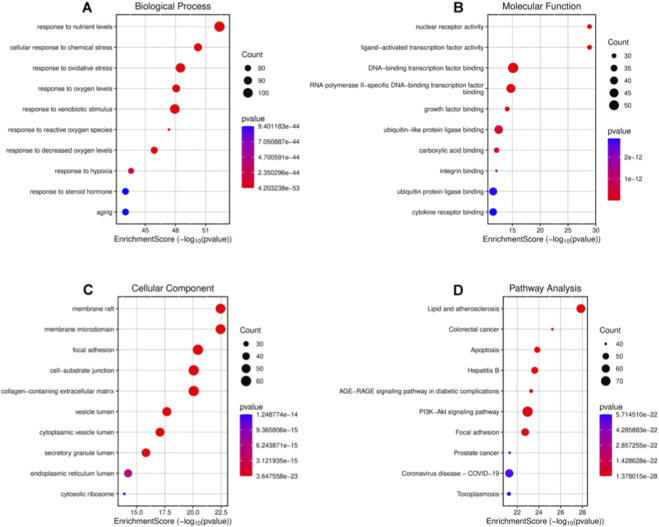
GO and KEGG enrichment analysis. GO analysis -BP **(A)**. GO analysis -MF **(B)**. GO analysis -CC **(C)**. KEGG analysis **(D)**.

In the BP category, the enriched terms were mainly associated with response to nutrient levels, cellular response to chemical stress, response to oxidative stress, response to oxygen levels, response to xenobiotic stimulus, response to reactive oxygen species, response to decreased oxygen levels, response to hypoxia, response to steroid hormone, and aging. These biological processes suggest that the shared targets are highly related to environmental stress adaptation, redox imbalance, endocrine response, and hypoxic signaling, all of which are relevant both to BPA toxicity and glioma progression (Figure 4A).

In the MF category, the common targets were mainly enriched in nuclear receptor activity, ligand-activated transcription factor activity, DNA-binding transcription factor binding, RNA polymerase II-specific DNA-binding transcription factor binding, growth factor binding, ubiquitin-like protein ligase binding, carboxylic acid binding, integrin binding, ubiquitin protein ligase binding, and cytokine receptor binding. These results indicate that the intersecting genes are involved in transcriptional regulation, receptor-mediated signaling, ligand recognition, and protein turnover, which may contribute to the broad regulatory influence of BPA on glioma-related signaling networks (Figure 4B).

In the CC category, enriched terms included membrane raft, membrane microdomain, focal adhesion, cell-substrate junction, collagen-containing extracellular matrix, vesicle lumen, cytoplasmic vesicle lumen, secretory granule lumen, endoplasmic reticulum lumen, and cytosolic ribosome (Figure 4C). These cellular component annotations suggest that the shared targets are localized not only in intracellular signaling compartments but also in membrane-associated and extracellular microenvironment-related structures, supporting a role for BPA in modulating both intracellular signal transduction and tumor microenvironment interactions.

### 3.5 KEGG pathway enrichment analysis

To further investigate the signaling pathways potentially involved in BPA-associated glioma, KEGG pathway enrichment analysis was performed on the 696 intersecting genes. A total of 204 significantly enriched pathways were identified (p.adjust < 0.05), indicating that the shared targets participate in a wide range of biological and disease-related signaling cascades.

KEGG enrichment analysis identified multiple pathways associated with cancer-related stress response, apoptosis, inflammatory signaling, cell adhesion, and survival regulation. Although several disease-label pathways, such as Hepatitis B and Coronavirus disease-COVID-19, appeared among the statistically enriched terms, these pathways were not interpreted as disease-specific mechanisms in glioma. Instead, they were considered to reflect shared molecular modules, including cytokine signaling, inflammatory response, apoptosis, oxidative stress, and PI3K-Akt-associated regulation. Therefore, we further prioritized pathways with direct relevance to glioma progression and cancer biology, including PI3K-Akt signaling, apoptosis, focal adhesion, AGE-RAGE signaling, oxidative stress-related processes, hypoxia-related response, and inflammatory cytokine signaling. After cancer-relevance re-ranking, the major functional modules remained centered on survival signaling, apoptosis resistance, inflammatory regulation, redox imbalance, hypoxia adaptation, and adhesion/migration-related pathways (Table 1). Although some enriched pathways are disease-category annotations, they reflect shared molecular mechanisms such as inflammatory response, survival signaling, stress adaptation, and cell adhesion remodeling.

**TABLE 1:** Functional re-annotation of enriched pathways according to glioma-relevant biological mechanisms and cancer hallmark categories.

Enriched pathway/module	Original KEGG term	Cancer/glioma-relevant interpretation	Related hub genes	Cancer hallmark category
PI3K-Akt signaling	PI3K-Akt signaling pathway	Survival, proliferation, apoptosis resistance	AKT1, IL6, TP53, MTOR	Sustained proliferative signaling/survival
Apoptosis	Apoptosis	Cell death resistance and stress response	TP53, CASP3, BCL2	Resistance to cell death
Focal adhesion	Focal adhesion	Adhesion, migration, invasion, ECM interaction	AKT1, MMP9, CTNNB1	Invasion and metastasis
Oxidative stress response	GO: Response to oxidative stress/ROS	Redox imbalance and toxicant response	TNF, IL6, TP53, STAT3	Oxidative stress adaptation
Hypoxia response	GO: Response to hypoxia/oxygen levels	HIF1α-related adaptation in glioma microenvironment	STAT3, AKT1, IL6	Hypoxia adaptation
Xenobiotic response	GO: Response to xenobiotic stimulus	BPA-related environmental toxicant response	ESR1, PPARG, TP53, AKT1	Environmental stress response
Inflammatory signaling	Hepatitis B/COVID-19-related shared genes	Shared cytokine/NF-κB/STAT inflammatory modules, not infection-specific interpretation	TNF, IL6, NFKB1, STAT3	Tumor-promoting inflammation

Of particular interest, the PI3K-Akt signaling pathway and apoptosis pathway were prominently enriched, suggesting that BPA may influence glioma progression by modulating cell survival, proliferation, anti-apoptotic signaling, and metabolic adaptation. Enrichment of the focal adhesion pathway further indicates that BPA-related targets may participate in cell adhesion, migration, and extracellular matrix interaction, all of which are closely linked to glioma invasiveness. In addition, pathways associated with inflammatory and oxidative stress responses support the hypothesis that BPA contributes to glioma biology through coordinated perturbation of tumor-promoting signaling networks. The top 10 enriched KEGG pathways are visualized in Figure 4D.

### 3.6 Molecular docking analysis of BPA with key hub proteins

To evaluate the potential direct interaction between BPA and representative hub proteins, molecular docking was performed using CB-Dock2. Based on the PPI results and topological importance, five core proteins were selected for docking analysis: STAT3 (PDB ID: 6NJS), AKT1 (PDB ID: 2UZR), TNF (PDB ID: 1JH5), IL6 (PDB ID: 5SFK), and TP53 (PDB ID: 1GZH). These targets were chosen because they ranked highly in the hub-gene analysis and are strongly associated with inflammation, survival signaling, transcriptional control, and glioma progression.

The docking results showed that BPA exhibited favorable binding affinities with all five selected proteins, with predicted binding energies ranging from −6.1 to −7.8 kcal/mol. The results are shown in Table 2. Negative Vina scores indicate favorable predicted ligand-protein interactions within the scoring model, and values in this range suggest moderate binding compatibility. These results suggest that BPA may have potential structural compatibility with several key proteins in the glioma-associated target network. However, the moderate docking scores do not provide direct evidence of biological binding or target engagement.

**TABLE 2:** Molecular docking results.

Target	PDB ID	Binding energy (kcal/mol)	Key interacting residues
AKT1	2UZR	−6.1	Thr21, Leu52
IL-6	5SFK	−7.8	Thr740, Gln743
STAT3	6NJS	−6.6	His437, Val490
TNF	1JH5	−7.4	Val77, His77
TP53	1GZH	−6.4	Arg1746, Ser1749

**FIGURE 5:**
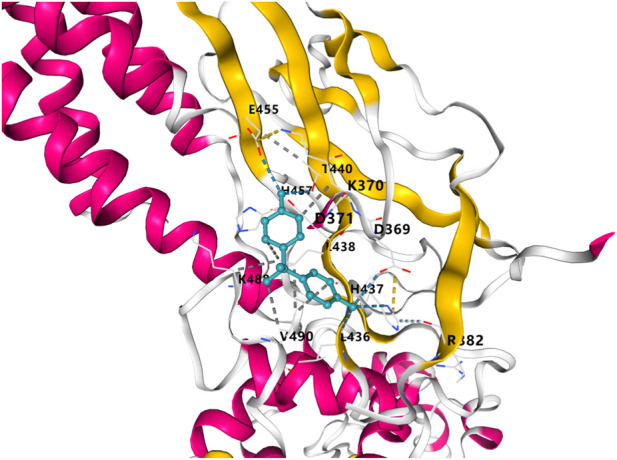
Molecular docking results of BPA with STAT3.

**FIGURE 6:**
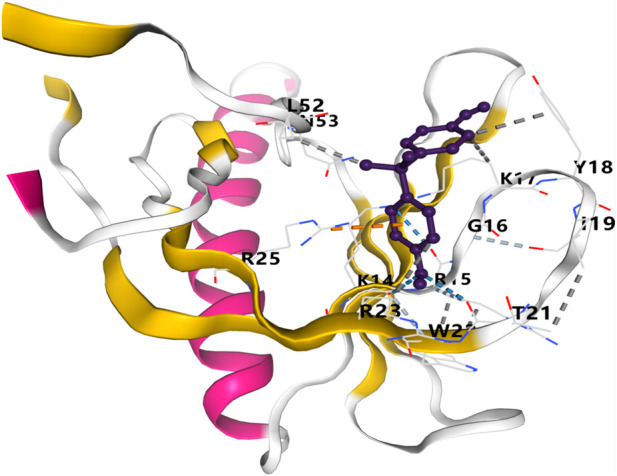
Molecular docking results of BPA with AKT1.

**FIGURE 7:**
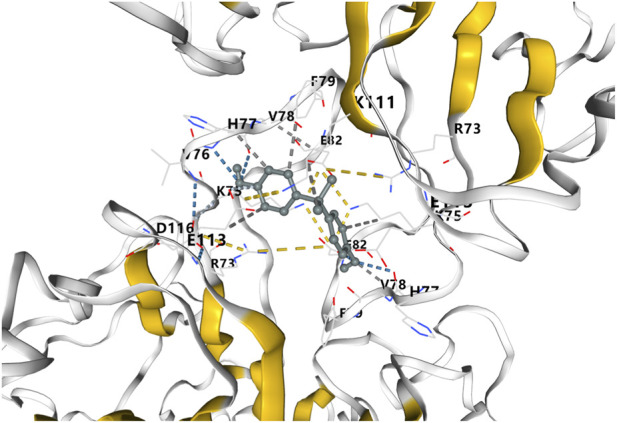
Molecular docking results of BPA with TNF.

**FIGURE 8:**
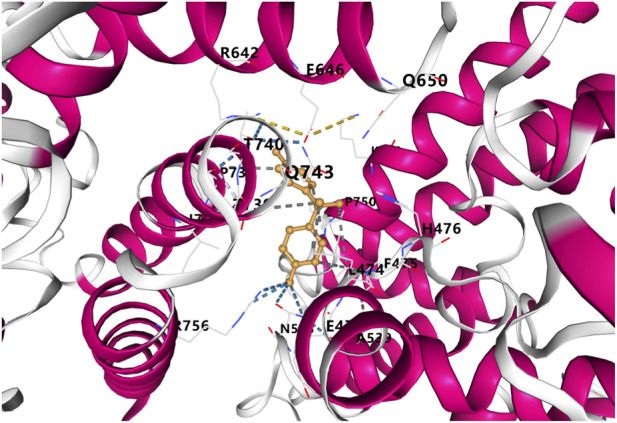
Molecular docking results of BPA with IL6.

**FIGURE 9:**
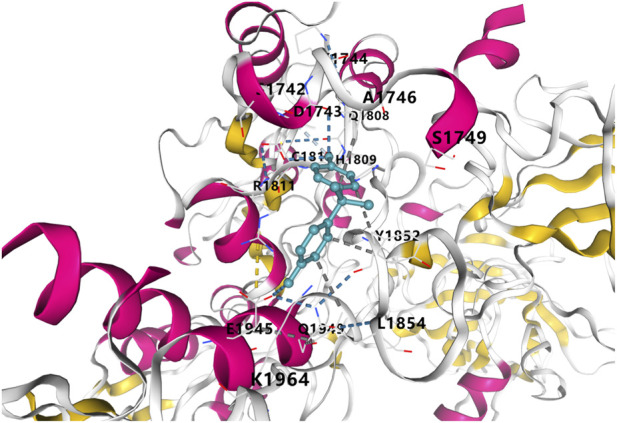
Molecular docking results of BPA with TP53.

Among the five proteins, the docking patterns supported stable ligand–protein interactions and provided structural evidence consistent with the bioinformatics findings. The ability of BPA to bind major regulatory proteins such as STAT3 and AKT1 is particularly noteworthy, because these molecules occupy central positions in pathways related to inflammation, proliferation, cell survival, and therapeutic resistance. Likewise, the predicted interactions with TNF and IL6 support a possible role of BPA in modulating inflammatory signaling, whereas binding to TP53 suggests a potential influence on stress-response and apoptosis-related pathways. Altogether, the molecular docking analysis reinforced the plausibility of the network toxicology predictions and further highlighted these proteins as candidate mediators of BPA-induced glioma progression. The docking visualizations are shown in Figures 5–9.

## 4 Discussion

The present study was designed to clarify the molecular basis by which bisphenol A (BPA) may contribute to glioma biology through an integrated network toxicology framework. By intersecting BPA-related targets with glioma-associated genes, prioritizing hub nodes in the protein–protein interaction network, validating their transcriptional relevance in an external dataset, and assessing ligand–protein binding feasibility, we identified a coherent set of inflammation-, survival-, and stress-response regulators that may mediate the link between environmental BPA exposure and glioma progression. These findings are important because they shift the discussion from single-target toxicity toward a systems-level model in which BPA is connected to glioma through coordinated perturbation of oncogenic and microenvironmental pathways rather than through an isolated receptor event alone.

A notable finding of this study was the identification of 696 shared targets between BPA and glioma, followed by the emergence of hub genes such as STAT3, AKT1, TNF, IL6, TP53, JUN, NFKB1, MTOR, MYC, PTEN, and TGFB1. This architecture is biologically plausible. Glioma progression is driven by highly interconnected signaling networks that integrate proliferation, apoptosis resistance, inflammatory cytokine signaling, hypoxic adaptation, and immune evasion. Similarly, BPA has been reported to exert pleiotropic effects on oxidative stress, steroid hormone signaling, inflammatory cascades, and cancer-related transcriptional programs (Cantonwine et al., 2010; Drzewiecki et al., 2023; Zhang R. et al., 2025). The broad overlap observed here therefore supports the idea that BPA-related tumor effects are more likely to arise from cumulative network perturbation than from a single linear pathway. In this context, the centrality of STAT3 and AKT1 is especially informative because these nodes sit at the intersection of cytokine signaling, metabolic adaptation, and treatment resistance, all of which are core hallmarks of aggressive glioma (Jia et al., 2025; Cheng et al., 2026; Dell'Albani et al., 2026; Linder et al., 2019).

The hub-gene validation step further strengthens this interpretation. Most of the top-ranked genes showed significantly higher expression in glioma stem cell samples than in normal neural stem cell controls, indicating that the network-derived candidates were not merely computational artifacts but were transcriptionally relevant in a biologically aggressive cellular context. The predominance of STAT3, AKT1, TNF, and IL6 among the highest-ranked hubs aligns well with current understanding of glioma as a disease sustained by reciprocal interactions between malignant cells and an inflammatory tumor microenvironment. STAT3 is widely recognized as a major regulator of glioma cell plasticity, mesenchymal transition, immune suppression, and therapeutic resistance, while IL-6 is a principal upstream activator of STAT3 and a marker of adverse outcome in diffuse glioma (Chen S. et al., 2026; Sun et al., 2026; Chen J. et al., 2026; Postlmayr et al., 2026). AKT1 and mTOR participate in survival signaling and metabolic reprogramming, whereas TNF and NFKB1 reinforce a chronic inflammatory state that supports invasion and immune remodeling (Mengnan et al., 2023; Wei et al., 2023). The comparatively distinct behavior of TP53 in the validation dataset is also noteworthy. Rather than weakening the model, this finding is consistent with the context-dependent nature of TP53 in glioma, where mutational status, transcriptional compensation, and post-translational regulation may uncouple expression abundance from canonical tumor-suppressive function.

The enrichment results provide a mechanistic bridge between the hub-gene architecture and the known biological effects of BPA. To improve the biological interpretation of the enrichment results, we focused on cancer- and glioma-relevant signaling modules rather than interpreting all KEGG disease-label terms literally. Some enriched KEGG terms, such as Hepatitis B and Coronavirus disease-COVID-19, are not directly related to glioma as diseases. Their enrichment likely reflects shared molecular components involved in cytokine signaling, inflammatory activation, apoptosis, oxidative stress, and PI3K-Akt regulation. Therefore, these terms were treated as indirect indicators of common stress and inflammatory modules rather than as glioma-specific pathways. Oxidative stress and redox imbalance may represent important mechanistic bridges between BPA exposure and glioma-associated biological processes. In the present study, GO enrichment analysis showed that the overlapping BPA–glioma targets were significantly enriched in response to chemical stress, response to oxidative stress, response to reactive oxygen species, response to oxygen levels, response to decreased oxygen levels, and response to hypoxia. These findings are consistent with the recognized ability of environmental toxicants to disturb redox homeostasis and induce cellular injury. For example, potassium bromate has been shown to induce oxidative damage and nephrotoxicity in rats, which can be ameliorated by vitamin C treatment (Nazir et al., 2026a). Ahmad et al. also demonstrated that potassium bromate causes cell lysis and oxidative stress in human erythrocytes (Ahmad et al., 2014). More recently, vitamin C was reported to regulate redox homeostasis and mitigate potassium bromate-induced oxidative injury in rat intestine (Nazir et al., 2026b). Although these studies were not conducted in BPA or glioma models, they provide relevant mechanistic evidence that environmental toxicant-induced oxidative stress can contribute to tissue injury through disruption of antioxidant defense and redox balance. Therefore, the enrichment of oxidative stress- and hypoxia-related pathways in our BPA–glioma target network supports the possibility that BPA may influence glioma progression by modulating redox-sensitive inflammatory, apoptotic, and survival-related signaling pathways.

The overrepresentation of responses to chemical stress, oxidative stress, reactive oxygen species, oxygen deprivation, hypoxia, steroid hormone signaling, and xenobiotic stimuli indicates that the shared target set captures precisely those cellular programs expected to respond to a persistent endocrine-disrupting toxicant in a hypoxic brain tumor ecosystem. Glioma cells are known to exploit oxidative and hypoxic stress pathways to maintain stemness, adapt to metabolic constraints, and resist therapy (Yuan et al., 2025; Brock et al., 2026). BPA, in turn, has been shown to alter redox homeostasis, hormone-responsive transcription, and inflammatory signaling in multiple disease models (Abd Elkader and Al-Shami, 2025; Nencini et al., 2026). The enrichment of membrane rafts, focal adhesions, extracellular matrix-associated compartments, and cytokine receptor binding further suggests that BPA-related effects may extend beyond intracellular stress to cell–cell communication, adhesion remodeling, and microenvironmental crosstalk. These observations are concordant with the KEGG results, particularly the enrichment of apoptosis, PI3K-Akt signaling, focal adhesion, AGE-RAGE signaling, and cancer-related pathways, all of which converge on survival, migration, and inflammatory signaling axes already implicated in glioma pathogenesis.

Among the enriched pathways, PI3K-Akt signaling deserves special attention. This pathway is one of the most consistently dysregulated cascades in glioma and functions as a master controller of growth, metabolism, survival, and treatment adaptation (Zhang Q. et al., 2026; Ma et al., 2026). Its identification in the present study not only supports the biological validity of the intersected target set, but also offers a mechanistic explanation for how BPA exposure could facilitate glioma progression. Recent functional studies indicate that BPA and related bisphenol compounds may promote tumor-associated phenotypes through ROS-, HIF-1alpha/VEGF/PI3K/AKT-, MAPK-, and IL-6/JAK2/STAT3-related signaling (Niu et al., 2024; Xia et al., 2022; Ptak et al., 2014; Wang et al., 2023; Zander et al., 2002). Our results therefore extend these observations by suggesting that PI3K-Akt activation is not an isolated downstream event, but part of a broader BPA-responsive network that includes IL6/STAT3, TNF/NF-κB, apoptosis regulators, and extracellular signaling components. This integrative view is a key innovation of the present study because it explains why exposure-related effects may persist even when no single pathway appears sufficient on its own.

The molecular docking results provide preliminary structural plausibility for the network and enrichment findings. BPA showed moderate predicted binding energies with STAT3, AKT1, TNF, IL6, and TP53, with docking scores ranging from approximately −6.1 to −7.8 kcal/mol. These values indicate possible binding compatibility but are below the level typically expected for strong binders. Therefore, the docking results should be interpreted cautiously as a target-prioritization reference rather than as evidence of direct biochemical binding. Importantly, docking should not be interpreted as proof of biological binding *in vivo*; rather, it provides a biophysical rationale for prioritizing targets for subsequent experimental validation. In the present context, the docking results are most valuable because they converge with the hub ranking and transcriptomic validation. When a protein is simultaneously central in network topology, dysregulated in disease-relevant samples, mechanistically linked to enriched pathways, and structurally compatible with the ligand of interest, it becomes a stronger candidate mediator of exposure-associated pathogenic effects. This convergence is particularly compelling for STAT3 and AKT1, which are not only topological hubs here but are also repeatedly implicated in glioma aggressiveness and therapeutic vulnerability in prior studies (Magalhaes et al., 2026; Yu et al., 2026; Liu et al., 2026).

Because the present study is based primarily on network toxicology, bioinformatics analysis, and molecular docking, the predicted BPA–glioma associations should not be interpreted as direct causal evidence. To avoid overstatement, the present findings are best understood as hypothesis-generating results that identify candidate molecular links requiring experimental validation (Niu et al., 2024). Nevertheless, several functional studies support the biological plausibility that BPA can regulate tumor-promoting phenotypes. In glioma models, BPA was reported to accelerate glioma cell proliferation in a time- and dose-dependent manner and to enhance glioma cell migration, invasion, and tumor growth (Xia et al., 2022). In colon cancer cells, BPA promoted migration and invasion through excessive ROS production and activation of the HIF-1α/VEGF/PI3K/AKT axis, whereas antioxidant intervention or HIF-1α knockdown reduced these effects (Ptak et al., 2014). In ovarian cancer cells, BPA stimulated proliferation, migration, invasion, and adhesion, partly through regulation of MMP-2, MMP-9, ICAM-1, MAPK, and PI3K/Akt signaling. In osteosarcoma, BPA promoted proliferation, migration, and invasion through DLGAP5-mediated activation of the IL-6/JAK2/STAT3 pathway (Wang et al., 2023). These functional observations are consistent with our enrichment results showing involvement of oxidative stress, hypoxia response, apoptosis, focal adhesion, inflammatory signaling, and PI3K-Akt signaling, as well as with the identification of STAT3, AKT1, IL6, TNF, and TP53 as candidate hub targets. However, direct experimental studies using glioma-specific models and exposure-relevant BPA concentrations are still required to validate whether these pathways mediate BPA-associated effects in glioma.

Experimental work published recently showed that BPA accelerates glioma cell proliferation and tumor growth, supporting the premise that BPA is not biologically inert in glioma models. Other studies on bisphenol analogs reported enhanced glioblastoma progression through PI3K/AKT/mTOR-centered mechanisms, suggesting that the bisphenol family may converge on common oncogenic signaling logic. In parallel, the glioma literature has increasingly emphasized the roles of STAT3, IL-6, and inflammation-driven signaling in mesenchymal transition, immune suppression, and therapeutic resistance (Chen et al., 2025; Zheng et al., 2026). Our study integrates these otherwise dispersed observations into a single analytical framework and proposes a mechanistic axis in which BPA is linked to glioma through inflammatory cytokines, oxidative stress, hypoxia adaptation, and survival signaling. The fact that our top hubs overlap with prior prognostic and mechanistic studies of glioma supports the robustness of the target-prioritization strategy. At the same time, the present study differs from earlier work by moving beyond a purely phenotypic or single-pathway interpretation and by combining target prediction, network topology, transcriptomic validation, functional annotation, and docking in a unified workflow.

These findings have several implications. At the conceptual level, they support the view that environmental endocrine disruptors should be considered within glioma research not merely as background exposures, but as candidate modulators of tumor signaling ecology (Li et al., 2024; Sheng et al., 2019; Qiao et al., 2025). At the translational level, the identified hubs may provide a rational starting point for biomarker development and exposure-informed therapeutic studies. For example, a BPA-responsive signature centered on STAT3/IL6/AKT1/TNF-related signaling could be explored in patient biospecimens, while experimental models could test whether BPA exposure amplifies glioma stemness, invasion, or therapy resistance through these axes. The enrichment of inflammatory and focal-adhesion pathways also suggests possible intersections with the tumor microenvironment, including macrophage recruitment, extracellular matrix remodeling, and cytokine-mediated immune polarization. Such interactions are clinically relevant because neuroinflammation and tumor-associated macrophages are now recognized as central determinants of glioblastoma progression and treatment failure.

Several limitations should be acknowledged. First, this was a bioinformatics- and docking-based study, and therefore causality cannot be established. The inferred BPA–target relationships require confirmation in cell-based and *in vivo* systems using exposure conditions relevant to human biology. Second, the external validation relied on GSE41031, which contained a very small number of samples, and this limits statistical power as well as generalizability. Third, target acquisition depended on public databases with different curation principles, which may introduce bias toward well-studied genes. Fourth, transcript abundance does not fully capture pathway activity, especially for proteins such as TP53 and STAT3 whose function depends heavily on mutation status or post-translational modification. Fifth, molecular docking estimates binding feasibility but cannot substitute for biochemical affinity assays, target engagement studies, or structural validation. Sixth, the selection of the top 20 hub genes was based on MCC ranking and should be regarded as a target-prioritization strategy rather than an absolute biological cutoff. Although MCC is a robust algorithm for identifying essential nodes in biological networks, alternative centrality methods, such as degree, closeness, and betweenness centrality, may generate partially different rankings. In addition, the GSE41031 validation dataset contained only three glioma stem cell samples and three normal neural stem cell samples, which limits statistical power and may increase the influence of outliers. The expression signals may also be affected by glioma subtype, tumor cellularity, cancer stem cell heterogeneity, culture conditions, and other confounders. Therefore, further validation using larger datasets and multiple network-ranking strategies is warranted. Seventh, the relatively large number of overlapping BPA–glioma targets is another limitation of this study. Although the inclusive target collection strategy was useful for exploratory network toxicology analysis, the 696 overlapping genes should not be interpreted as a highly specific BPA–glioma signature. This target set may include low-confidence predicted targets, broadly expressed stress-response genes, and pan-cancer-related molecules. Therefore, the present findings should be regarded as hypothesis-generating. Future studies should refine this target network by applying stricter confidence-based criteria, such as retaining targets supported by experimental evidence, requiring consensus across multiple target databases, or applying prediction-score thresholds. Such filtering, together with experimental validation, will be necessary to identify high-confidence BPA-associated targets in glioma. Eighth, the molecular docking analysis also has limitations. Although BPA showed negative docking scores with selected hub proteins, the predicted binding energies were moderate rather than strong. Docking scores alone cannot confirm true biochemical binding, target engagement, or functional regulation in cells. In addition, the present docking analysis did not include systematic pose-clustering analysis, comprehensive two-dimensional interaction maps for all major poses, or benchmarking against known ligands of the same proteins. Future studies should incorporate these analyses, together with molecular dynamics simulations, binding affinity assays, cellular thermal shift assays, or other target-engagement experiments, to confirm whether BPA directly interacts with these candidate proteins under biologically relevant conditions. These limitations, however, do not negate the value of the present work; rather, they define a clear agenda for follow-up studies, including dose-controlled BPA exposure experiments in glioma stem-like models, phosphoproteomic validation of STAT3/AKT signaling, orthogonal verification in larger patient cohorts, and integration of exposure measurements, candidate core targets, and clinicopathologic annotations. In addition, although the shared BPA–glioma targets were closely related to cancer-associated mechanisms, including inflammation, oxidative stress, hypoxia adaptation, apoptosis regulation, focal adhesion, and PI3K-Akt signaling, these molecular features are not specific indicators of glioma development. Many of the identified hub genes are broadly involved in multiple malignancies. However, because all validation samples used in the present study were derived from glioma-related tissues or cells, the observed molecular alterations are most likely relevant to glioma-associated biological processes. Further glioma-specific experimental validation is therefore necessary to determine whether these common cancer-related targets mediate BPA-associated effects in glioma. Therefore, the present study should be interpreted as a preliminary bioinformatics and network toxicology analysis that generates testable hypotheses regarding the potential involvement of BPA in glioma-related molecular networks. Direct experimental validation is required before any causal relationship between BPA exposure and glioma initiation or progression can be established.

Although STAT3, AKT1, TNF, IL6, and TP53 were identified as central nodes in the BPA–glioma interaction network, these genes are not glioma-specific biomarkers and are broadly involved in multiple malignancies. Therefore, their identification should not be interpreted as direct evidence that BPA specifically induces glioma onset or progression. Rather, these molecules may represent common oncogenic and microenvironment-related signaling nodes through which BPA could potentially influence glioma-associated biological processes. Future studies using glioma-specific models, patient-derived samples, and exposure-based validation are required to clarify whether BPA exerts tumor-type-specific effects in glioma.

In summary, this study supports a model in which BPA may contribute to glioma progression through a coordinated network centered on inflammatory signaling, oxidative and hypoxic stress responses, apoptosis regulation, and PI3K-Akt-associated survival programs. By integrating target prediction, network analysis, transcriptomic validation, enrichment profiling, and molecular docking, our work provides a mechanistically informed foundation for future experimental studies aimed at clarifying how environmental BPA exposure intersects with glioma biology and whether this interaction can be leveraged for risk stratification or therapeutic intervention.

## 5 Conclusion

In conclusion, this study integrated network toxicology, bioinformatics validation, enrichment analysis, and molecular docking to explore the potential molecular links between BPA and glioma progression. A broad set of overlapping BPA–glioma targets was identified, and several core candidate mediators, including STAT3, AKT1, TNF, IL6, and TP53, were prioritized through PPI network analysis. Functional enrichment analysis indicated that these targets were mainly associated with oxidative stress, hypoxia response, xenobiotic response, inflammatory signaling, apoptosis regulation, focal adhesion, and PI3K-Akt-associated survival pathways. Molecular docking further provided preliminary structural plausibility for the interaction between BPA and representative hub proteins.

These findings suggest that BPA-related targets may be involved in glioma-associated inflammatory, stress-response, and survival-related signaling networks. Importantly, the identified hub genes and enriched pathways are not glioma-specific biomarkers and are also involved in the pathogenesis of multiple malignant tumors. Therefore, the present findings should not be interpreted as direct evidence that BPA specifically causes glioma initiation or progression. Rather, because the validation dataset and biological context used in this study were derived from glioma-related samples, these molecular effects may be particularly relevant to glioma biology and may provide a mechanistic basis for future investigation.

Overall, this study provides a systems-level and hypothesis-generating framework for understanding how BPA exposure may intersect with glioma-related molecular networks. Further experimental studies using glioma cell models, glioma stem-like cells, patient-derived samples, animal models, and exposure-relevant BPA concentrations are required to validate these candidate targets and clarify whether BPA contributes to glioma initiation, progression, invasiveness, or therapeutic resistance.

## Data Availability

The datasets analyzed in this study are publicly available. Gene expression validation data were obtained from the Gene Expression Omnibus under accession number GSE41031. BPA-related and glioma-related target information was obtained from public databases, including PubChem, PharmMapper, SwissTargetPrediction, CTD, GeneCards, OMIM, STRING, and UniProt. The original contributions presented in the study are included in the article/Supplementary Material; further inquiries can be directed to the corresponding author.
